# Phage susceptibility testing and infectious titer determination through wide-field lensless monitoring of phage plaque growth

**DOI:** 10.1371/journal.pone.0248917

**Published:** 2021-03-23

**Authors:** Prisca Perlemoine, Pierre R. Marcoux, Emmanuel Picard, Emmanuel Hadji, Marc Zelsmann, Grégoire Mugnier, Aurélie Marchet, Grégory Resch, Larry O’Connell, Eric Lacot

**Affiliations:** 1 Department of Microtechnologies for Biology and Health, LETI, CEA, University Grenoble Alpes, Grenoble, France; 2 SINAPS, PHELIQS, DEPHY, IRIG, DRF, CEA, University Grenoble Alpes, Grenoble, France; 3 LTM–Micro and Nanotechnologies for Health, CNRS, CEA, University Grenoble Alpes, Grenoble, France; 4 Department of Fundamental Microbiology, University of Lausanne, Lausanne, Switzerland; 5 SyMMES, IRIG, DRF, CEA, University Grenoble Alpes, Grenoble, France; 6 Laboratoire Interdisciplinaire de Physique, CNRS UMR 5588, University Grenoble Alpes, St Martin d’Hères, France; Universidade de Aveiro, PORTUGAL

## Abstract

The growing number of drug-resistant bacterial infections worldwide is driving renewed interest in phage therapy. Based on the use of a personalized cocktail composed of highly specific bacterial viruses, this therapy relies on a range of tests on agar media to determine the most active phage on a given bacterial target (phage susceptibility testing), or to isolate new lytic phages from an environmental sample (enrichment of phage banks). However, these culture-based techniques are still solely interpreted through direct visual detection of plaques. The main objective of this work is to investigate computer-assisted methods in order to ease and accelerate diagnosis in phage therapy but also to study phage plaque growth kinetics. For this purpose, we designed a custom wide-field lensless imaging device, which allows continuous monitoring over a very large area sensor (3.3 cm^2^). Here we report bacterial susceptibility to *Staphylococcus aureus* phage in 3 hr and estimation of infectious titer in 8 hr 20 min. These are much shorter time-to-results than the 12 to 24 hours traditionally needed, since naked eye observation and counting of phage plaques is still the most widely used technique for susceptibility testing prior to phage therapy. Moreover, the continuous monitoring of the samples enables the study of plaque growth kinetics, which enables a deeper understanding of the interaction between phage and bacteria. Finally, thanks to the 4.3 μm resolution, we detect phage-resistant bacterial microcolonies of *Klebsiella pneumoniae* inside the boundaries of phage plaques and thus show that our prototype is also a suitable device to track phage resistance. Lensless imaging is therefore an all-in-one method that could easily be implemented in cost-effective and compact devices in phage laboratories to help with phage therapy diagnosis.

## Introduction

The increasing number of drug-resistant bacterial infections is an emerging global health crisis that is driving a growing demand for phage therapy [1]. First implemented in 1919 [2], phage therapy is based on the use of highly specific bacterial viruses called bacteriophages or phages. Through a lytic life cycle, some phages are able to replicate within the cytoplasm of bacterial cells before being released through lysis of their host. In recent years, promising clinical studies of phage therapy were conducted to treat infection of burn wounds [3], urinary tract infections [4], and chronic otitis [5] caused by antibiotic-resistant bacteria. Currently, increasing evidence is leading to a consensus in the scientific community regarding synergism between phages and antibiotics [6, 7].

Regarding the high specificity of phages to particular bacterial species and even particular strains thereof, phage therapy is envisioned as part of a shift to a new paradigm of personalized medicine wherein personalized phage cocktails are administered combining only the most effective phages for a given bacterial isolate [8]. Accordingly, this approach requires access to large phage libraries (or phage banks) that must be maintained and constantly replenished with novel lytic phages targeting otherwise phage-resistant clones. Moreover, evolutionary selection of bacterial mutants resistant to the phages composing the cocktail should be taken into account, as much as possible, in the process of developing personalized phage cocktails and carefully monitored during treatment to avoid therapeutic failures [9]. Therefore, the success of personalized phage medicine largely relies on our capacity to quickly identify and isolate new lytic phages against a diverse range of pathogens [10] as well as to monitor the development of phage-resistance.

The classical method for phage discovery and isolation is to mix liquid cultures of clinical bacterial isolates with samples expected to contain phages, [11] *e*.*g*. human feces [12], chopped chicken intestines [13], raw sewage [14–16], farmyard slurries [17] and river waters [18]. After a suitable period and temperature of incubation, remaining host cells and debris are removed through centrifugation/filtration and the aqueous phase (i.e. the so-called *lysate*) is assayed for the presence of phages using the double agar overlay method [8]. At this stage, the lysate may contain several different phages that have been proliferated in, especially if samples contained a complex and diverse flora. For instance, Primrose *et al*. observed as many as seven different plaque morphologies in a double agar overlay assay using a single *Escherichia coli* HfrH strain as a host, and river samples collected downstream from the effluent discharge point of a sewage works as a source of novel phages [18]. To the best of our knowledge, despite the laborious nature of this process, direct visual evaluation of plaques by the experimenter is still the sole technique routinely used in laboratories to discriminate between phages. Usually this time-consuming visual inspection of phage plaque is only the first step of the phage characterization procedure and is combined with other evaluations, such as time-kill kinetics assay through kinetic optical density measurements [19]. Moreover, genome sequencing is performed on candidate viral strains for therapeutic use to exclude temperate phages and detect the presence of undesirable genes encoding antimicrobial resistance or virulence factors [20]. Accordingly, more automated approaches are highly desired, including tools that could quickly detect incipient plaques, accurately enumerate phage titer, study and discriminate between their morphologies and growth kinetics, and simultaneously detect phage-resistance selection. Indeed, such a tool would greatly help enrichment and screening of existing phage banks. While Koch [21]reported one of the first studies of plaque growth kinetics through direct visual measurement, other groups reported attempts to automate this assay through time-lapse photography [22–24]. These early attempts allowed the study of only up to six plaques simultaneously. In this work, we evaluate lensless imaging for the high-throughput study of plaque growth kinetics as well as the detection of phage-resistance selection.

Lensless imaging is inspired by the in-line holography technique of D. Gabor [25]. It consists of imaging a sample placed directly on top of an imaging sensor without the use of any intervening optical objectives. Lensless imaging presents several advantages. Firstly, there are no optical aberrations caused by the use of lenses. Moreover, because the sample is close to the sensor, the magnification is equal to one. The resolution and field of view (FoV) of the image is then solely limited by pixel pitch, FoV, and size of the sensor. Therefore, a wider field of view is accessible for monitoring of the sample, compared to that of conventional optical microscopy [26, 27]. Moreover, since the pixel pitch of modern sensors can be as low as a few microns, lensless imaging can resolve structures of a few dozens of microns, such as bacterial microcolonies [28]. Finally, compared to conventional optical microscopes, lensless prototypes are compact and cost-efficient devices that can easily fit into standard laboratory incubators.

The lensless imaging approach has already been implemented to detect and count viral plaques and study eukaryote cells death dynamics in plaques using eukaryotic viruses with a 24-mm^2^ FoV [29]. However, the authors do not provide results on the plaque growth kinetics. We therefore suggest using a similar approach but with a wider 3.3-cm^2^ FoV CMOS sensor to study phage plaques. Here we report computer-assisted detection and counting of phage plaques as well as simultaneous measurement of the growth kinetics of nineteen plaques from the same sample. Finally, we propose a lensless imaging technique to monitor phage-resistance selection through imaging of the interior of phage plaques.

## Materials and methods

### Bacteria and bacteriophages

For the study of plaque count and growth rate, the methicillin-susceptible *S*. *aureus* (MSSA) strain Laus102 –isolated from a healthy patient at the University Hospital of Lausanne–and the lytic phage vB_SauM_2002 –isolated from a wastewater plant in Vidy, Lausanne, Switzerland–were used. Phage vB_SauM_2002 belongs to the Myoviridae family and harbors 99.92% nucleotide sequence identity over 91% of its ca. 140Kb genome with phage Sb-1 (GenBank: HQ163896.1).

For the study of resistance detection, the clinical isolate *K*. *pneumoniae* strain R405 TMP-8 and the lytic phage vB_Kpn_5055 –isolated from the wastewater plant in Vongy, Thonon-les-bains, France–were used. The closest relative of vB_Kpn_5055 is the unclassified Przondovirus phage Kund-ULIP47 (GenBank: MK380015.1) with 98.40% nucleotide sequence identity over 97% of its ca. 41Kb genome.

Genomic phage DNA was purified following the traditional phenol/chloroform extraction protocol of the Center for Phage Technology of the Texas A&M University [30]. Purified DNA was sent to Eurofins Genomics Germany GmbH (Ebersberg, Germany) for sequencing. Phage genomic DNA fragment libraries were prepared with an optimized protocol and standard Illumina adapter sequences. Sequencing was performed with Illumina technology, NovaSeq 6000 (read mode 2 x 150bp).

For both bacteria and prior to the experiments, a loopful of 20% glycerol stock kept at -80°C was plated on a fresh Luria Bertani Broth (LB) agar plate and incubated aerobically at 37°C. Sixteen hours later, a single colony was resuspended in 5 mL fresh LB and incubated aerobically for 16 hr at 37°C with agitation (200rpm).

Phage solutions were prepared through simple phage amplification in liquid. Briefly, Bacterial cultures (Laus102 or R405 TMP-8) were grown at an OD_600nm_ of 0.3 before the corresponding phage were added at a multiplicity of infection (MOI) of 1 (vB_SauM_2002) or 0.01 (vB_Kpn_5055). The mixture was incubated aerobically at 37°C under 200 rpm agitation for 16 hr. After a centrifugation at 8000g for 15 min, the supernatant was filtered at 0.45 μm and titered using a soft-agar assay. Briefly, 250 μL of an overnight bacterial culture (Laus102 or R405 TMP-8) was mixed with serial dilutions of the corresponding phage solution and 5mL of molten soft-agar. The mixtures were poured in petri dishes and left at room temperature to allow the agar to solidify. Petri dishes were placed at 37°C for 16 hr and phage titers were determined by plaque counting. Phage solutions were stored at 4°C until further use. For the phage plaque counting experiment, 100μL of phage vB_SauM_2002 suspension was adjusted to 1.2×10^3^ PFU/ml and 250 μL of host bacteria *S*.*aureus* Laus102 culture are added to 5 mL of molten soft-agar. The mixture is smoothly shaken for homogenization and 2 mL are poured into a 35 mm diameter cell culture dish (Falcon® ref.353001). Finally, 1 mL of silicone oil AR 20 (Aldrich Chemistry ref. 10836-100ML) is added on top of the solidified soft agar layer. The silicon oil allows gas exchange to the growth medium while preventing it from drying.

To study the phage/bacteria interaction we choose the soft-agar approach over a culture in liquid broth since the former is a more faithful model of spatially structured environments in which most environmental bacteria reside (e.g. biofilms, soil, or plant and animal tissues) [31].

### Image acquisition and processing

#### Custom lensless prototype

**Fig 1:**
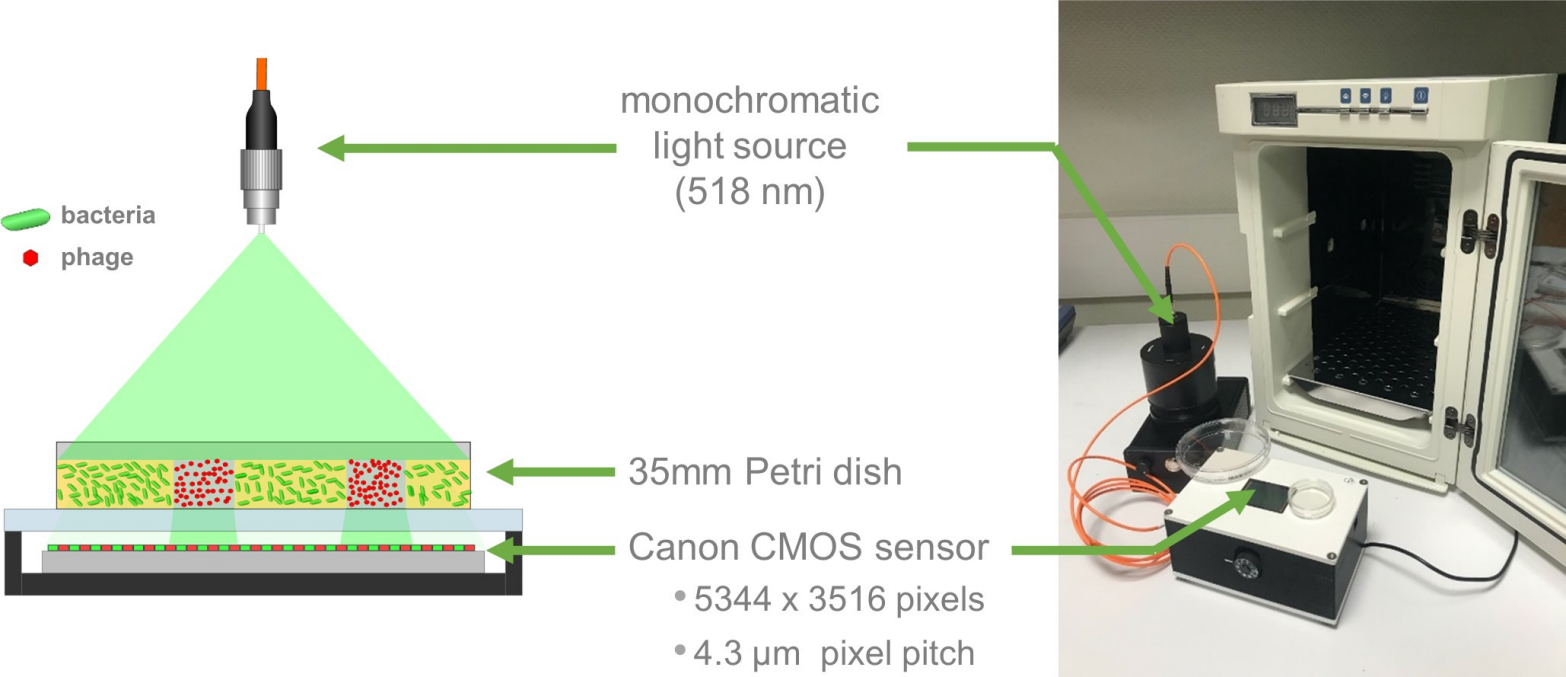
Customized lensless prototype. (a) Drawing and (b) picture of the lensless configuration used to measure phage plaque growth. The prototype is small enough to fit in a standard laboratory incubator (here a Heraterm 18L).

The lensless imaging system is shown in Fig 1. The apparatus is composed of a detection system and an illumination module. The detection system consists of a 22.3 × 14.9 mm^2^ complementary metal oxide semiconductor (CMOS) advanced photo system type-C (APS-C) sensor, repurposed from a Canon 1200D DSLR. The sensor–along with its accompanying supply and readout circuitry–is incorporated into a custom housing as shown in Fig 1. The sensor consists of an array of 5344 × 3516 pixels of 4.3 μm pitch. The Petri dish, prepared as indicated above, is placed directly on the sensor and illuminated from above by a light source. The light source consists of a monochromatic green LED (518 nm) coupled into a 200 μm core diameter multimode optical fiber (Thorlabs M72L02). Images are acquired using the built-in Canon software and converted from Canon native raw format (.CR2) to.tiff files using Dcraw software. The images are then processed using two different algorithms; the first processes the entire image area to detect plaques, while the second processes only a cropped sub-image of each plaque to compute the growth rate.

#### Computer-assisted plaque detection and counting

**Fig 2:**
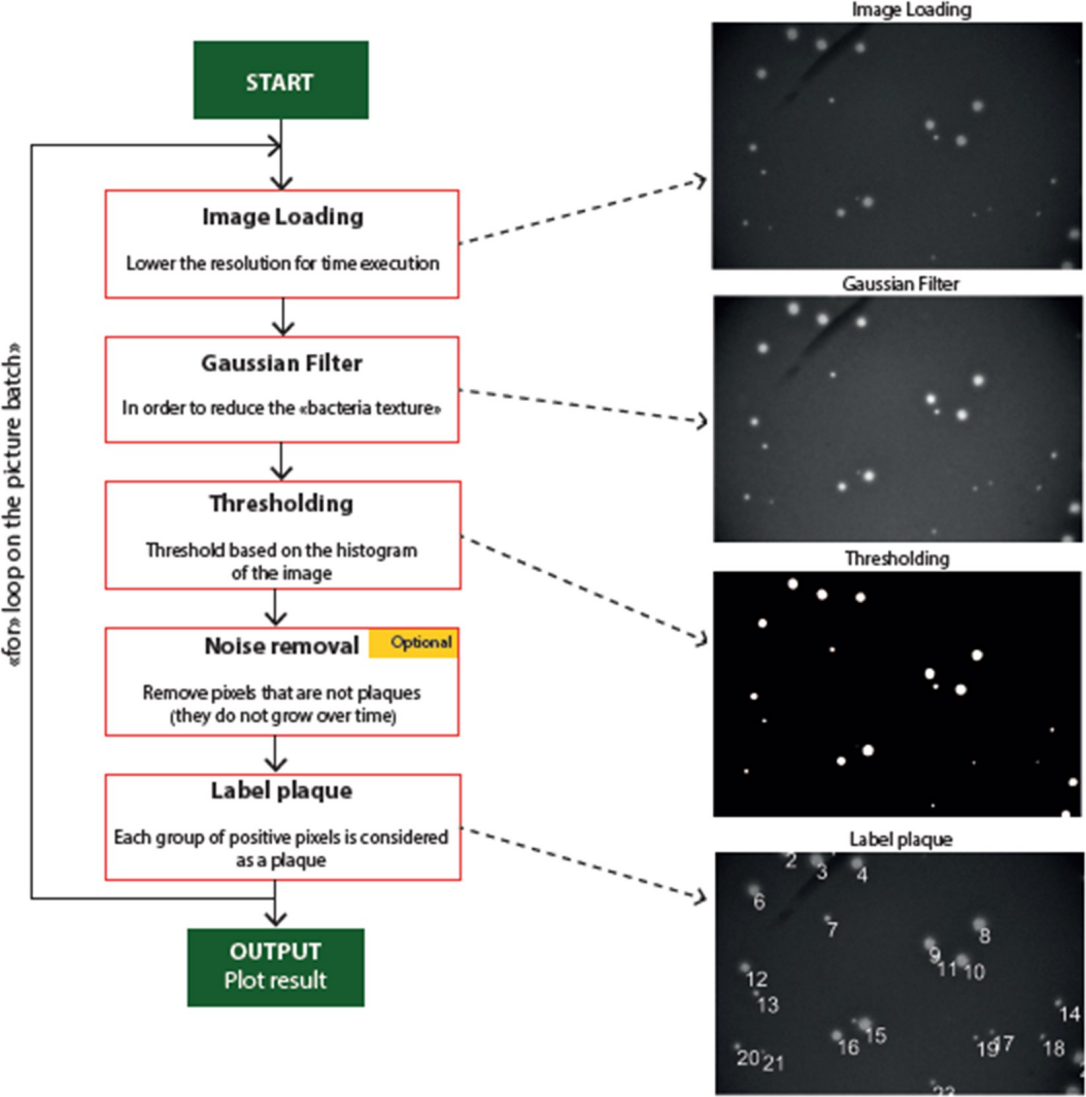
Algorithm structure to count phage plaques.

The plaque detection is performed using an in-house algorithm that uses a computer-assisted method to count the phage lysis plaques. Images are processed in order to isolate the shape of the plaque from the background, and then are labelled to yield the number of detected phage plaques (see Fig 2).

#### Plaque growth kinetics

**Fig 3:**
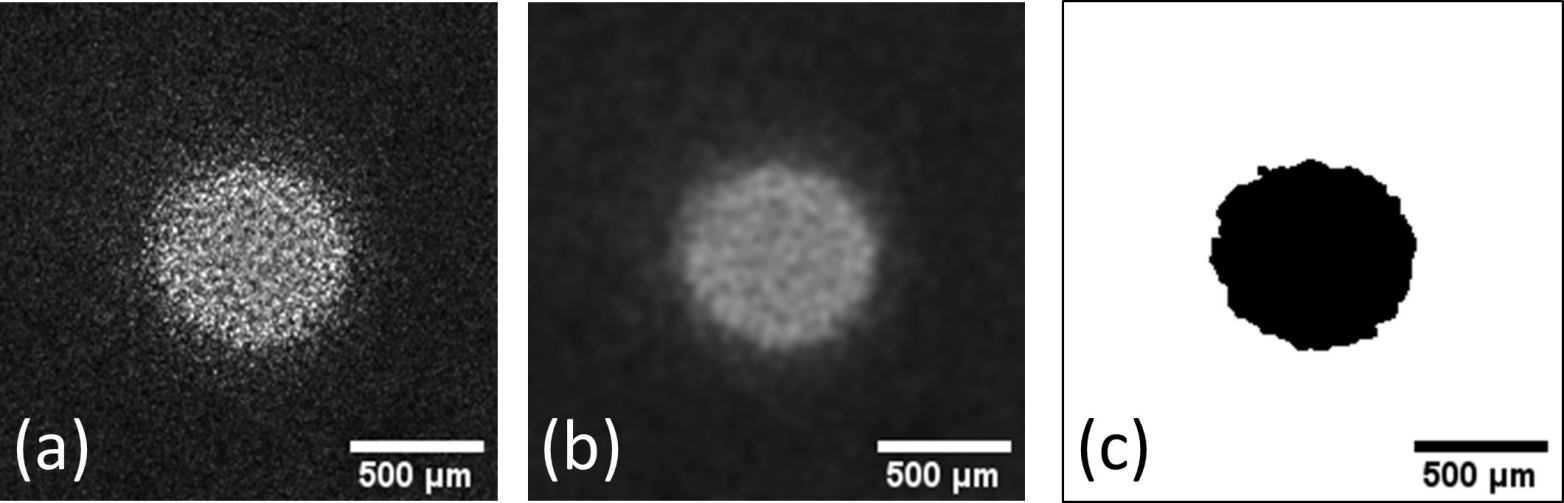
Image processing for plaque growth kinetics study. (a) Unprocessed phage plaque image (b) After a Gaussian blur filtering (c) Binarized final image.

A different algorithm is used to study the growth kinetics of phage plaques using the Fiji software package [32] (see S1 File) Each plaque is isolated and processed separately (see S1 Dataset for examples of phage plaque image stacks). First, a Gaussian blur filter with two-pixel radius is applied in order to remove high spatial frequency noise. Then the image is thresholded and binarized using the built-in Otsu algorithm (see Fig 3) [33]. The plaque area is finally retrieved using the ‘*Analyze particles’* module (see S2 Dataset). The plaque radius is computed using:

$$r=\sqrt{\frac{A}{\pi }}$$

The time-derivative of the phage plaque radius ($\frac{dr}{dt}$, expressed in μm/hr) was calculated in order to retrieve the radial velocity of phage plaques, referred to hereafter as the ‘plaque growth rate’. In the case of a plaque whose radius grows linearly with time, the plaque growth rate is determined with the slope of curve, *i*.*e*. a constant. Linear regression was performed using Origin from OriginLab.

## Results and discussion

### Plaque detection and counting

**Fig 4:**
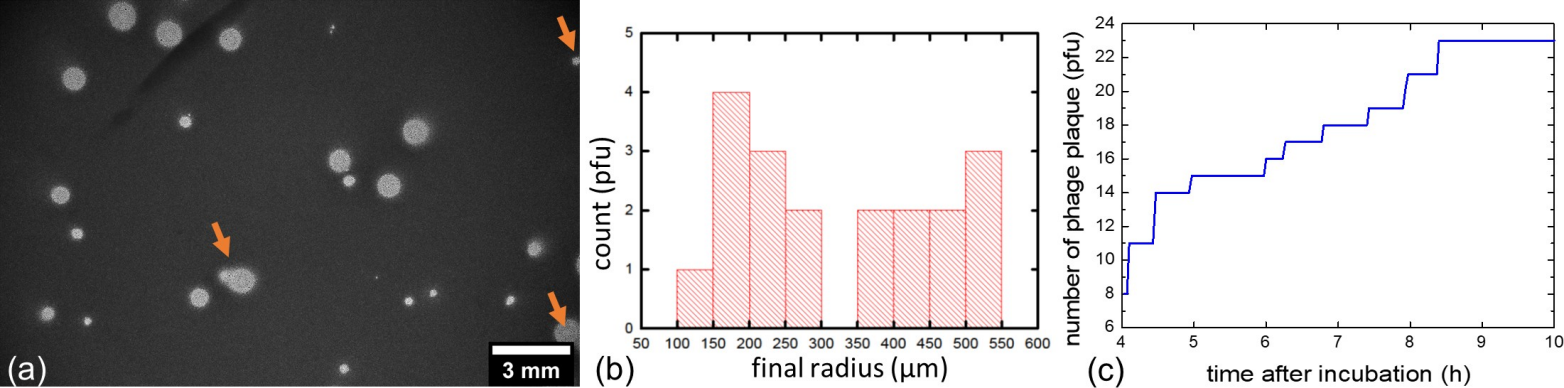
Acquisition of single-layer agar assay through lensless imaging. (a) Image of a single-layer soft agar assay after 21 hr 44 minutes of incubation at 37°C, with the dark background corresponding to the area of bacterial growth and bright disks to lysis plaques. The orange arrows indicate plaques that were not excluded from the growth kinetics study. (b) Corresponding plaque radius histograms and (c) cumulative graph of plaques detected as a function of time.

**Fig 5:**
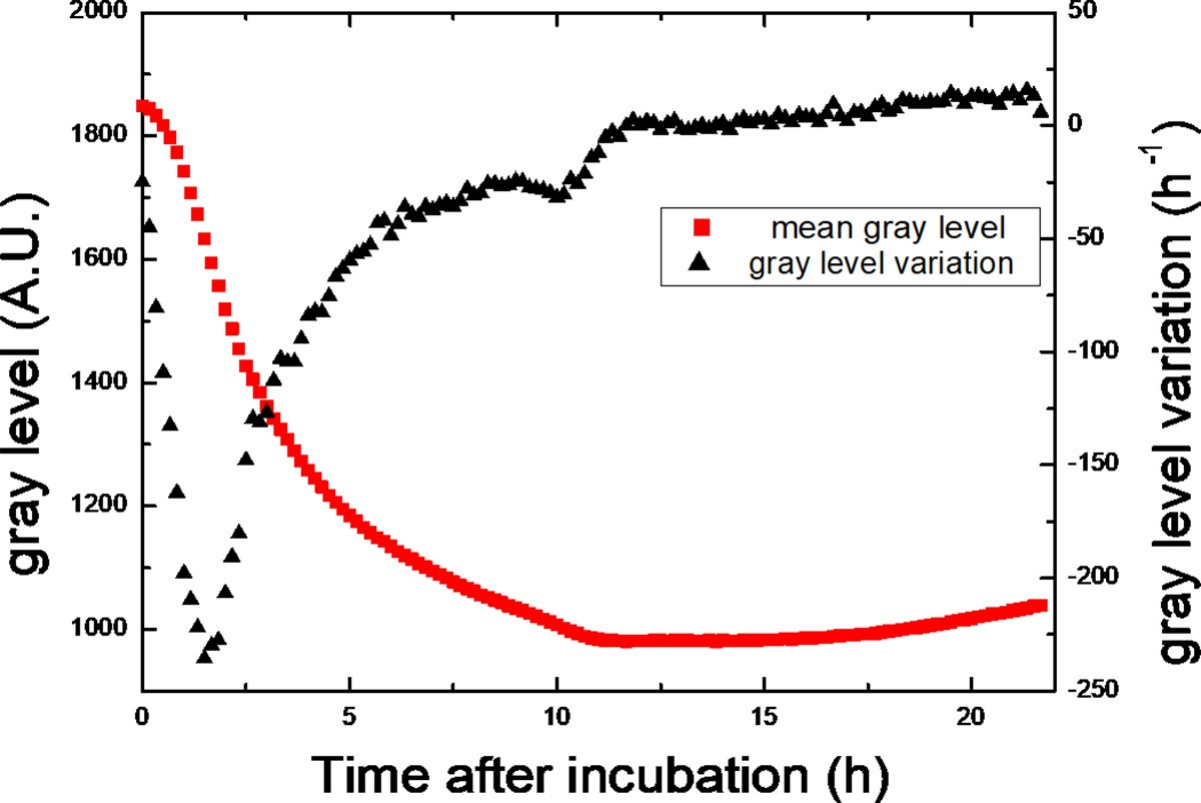
Evolution of the mean gray level (red curve) of bacterial lawn and its variation (black curve). The gray level variation is the derivative of the mean gray level. It highlights the evolution of the growth rate of the bacteria inside the lawn. It is maximal after 2 hr of incubation.

An image of a single-layer soft agar assay recorded after 21 hr 44 min of incubation at 37°C is shown in Fig 4 (A) along with the corresponding size distribution of plaques at this time point (b) and the number of plaques detected over time (c). A movie of the acquisition is also available as a (S1 Movie). The dark background on image (a) corresponds to the bacterial lawn while bright disks correspond to plaques. It is important to note that the background monotonically darkens during the first 10 hr of incubation as bacteria proliferate within the lawn, and then stabilizes at a constant gray level of around 1000 A.U. for the following 14 hr (Fig 5, red curve). Moreover, as indicated by the gray level variation maximum of -22.5 hr^-1^ (Fig 5, black curve), the bacteria within the lawn reached a maximum of exponential growth rate after 2 hr of incubation.

The first plaque was detected only 4 hr after the start of incubation (Fig 4C) indicating that the phage was indeed lysing the selected bacterial strain. After 8 hr 20 min of incubation, a total of 23 different plaques were counted within the FoV and this number did not further increase after the 10 hr mark, likely indicating that all plaques within the considered FoV did appear. Knowing the sensor and culture dish area (3.3 and 9.6 cm^2^, respectively), the volume of soft-agar in the dish (2 mL) and the volume of phage suspension introduced in the culture medium (100 μL), we estimated a phage titer of 1.7×10^3^ PFU/mL. This titer is only 30% different from the titer estimated with standard double layer agar assay. In other words, in our settings the continuous lensless monitoring of a single-layer soft agar assay can yield phage susceptibility results within 4 hr and phage titer in solution after only 8 hr 20 min. In contrast, the technique routinely used in laboratory requires around at least 12 hr of incubation and visual inspection of the agar plate to obtain similar data.

Of note, neither plaques at the edge of the FoV nor confluent plaques were considered, leaving us with 19 inidividual plaques. A great variability in the plaque radius of these 19 plaques was observed at time point 21 hr 44 min, ranging from 147 μm to 546 μm with a mean of 325 ± 140 μm.

### Study of plaque growth kinetics

**Fig 6:**
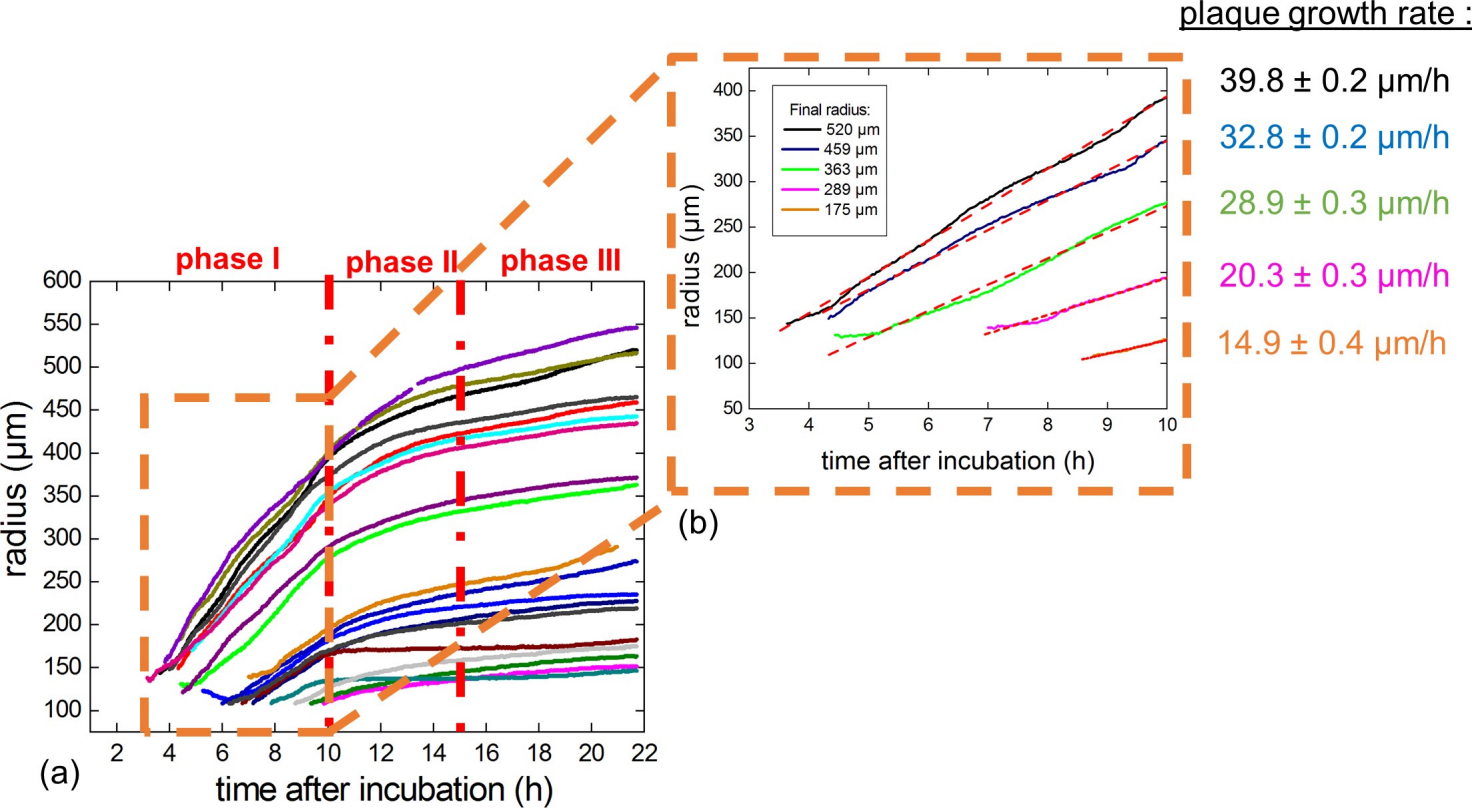
Growth of plaque radius over time. (a) **Plaque expansion on plates** expressed as μm radius as a function of time for the nineteen plaques considered. Larger plaques were those that started to grow earlier than smaller plaques. (b) **Representative examples of linear fits** of the curves obtained in the first 10 hr of incubation for plaques of various final radii. The slopes of the fitted curves correspond to the growth rate of the respective plaques.

*When isolating each plaque within the FoV (from Fig 4), we could detect and study the* growth rate of the first plaque already after 3 hr 30 min of incubation (Fig 6). Of note, the plaques first appearing at 3 hr 30 min hr grow to a radius of >400 μm by the end of the experiment (at 21 hr 44 min) whereas the last-appearing plaques appearing at 10 hr would not exceed a radius of 150 μm by the end of the image acquisition process. This likely reflects delayed adsorption of some phage vB_SauM_2002 on cells of its bacterial host *S*. *aureus* Laus102, with larger plaques resulting from phage particles that adsorbed early and smaller plaques resulting from phage particles that adsorbed later during the experiment [34].

We separated the plaque expansion curves into three distinct phases (Fig 6A).

#### Phase I

During the first 10 hr of incubation (phase I), the plaque growth, reflected by the increase in radius over time), was linear (Fig 6A). Therefore, we derived a constant growth rate for each plaque during phase I using the slopes of the linear fits (Fig 6B). As observed with a representative selection of five different plaques of final radii ranging between 175 to 520 μm (Fig 6B), the growth rates of the larger plaques were higher than the ones of the smaller plaques, highlighting that the plaque growth rates correlated with the final radius size of the plaques.

Linear growth of plaques has been reported previously [22–24], and was expected from theoretical models suggesting linear plaque growth rate $\frac{dr}{dt}$ dependent only on the phage diffusion rate *D* and the latency time *L* [21] following the equation:

$$\frac{dr}{dt}=10.\sqrt{\frac{D}{L}}$$

However, this overly simplistic model did not take into account the differing plaque growth rates in relation to plaque size and the surrounding bacterial density. More complex models demonstrated that a higher initial bacterial density inside the top-agar layer resulted in smaller plaques as well as lower plaque growth rates [35]. Moreover, as explained above, the phage adsorption rate contributes to differences in the final size of a plaque. Taken together, these parameters induce a variance in the timing of the first infection event for each individual phage. As observed in phase I, it might be assumed that the first infection event that produces a phage progeny happened with a significant delay in smaller plaques compared to larger plaques. Indeed, the smallest plaques were first observed on plates approximately 5 hr after the first large plaque was observed. According to this significant delay, a higher initial bacterial density surrounding smaller plaques might be assumed. Therefore, our experiments would support the previously observed correlation between a higher initial bacterial density around small plaques associated to a lower plaque growth rate [35]. The associated lower growth rate of late-appearing plaques would be further supported by the differences in plaque growth rates observed in phase I (Fig 6B).

#### Phase II

**Fig 7:**
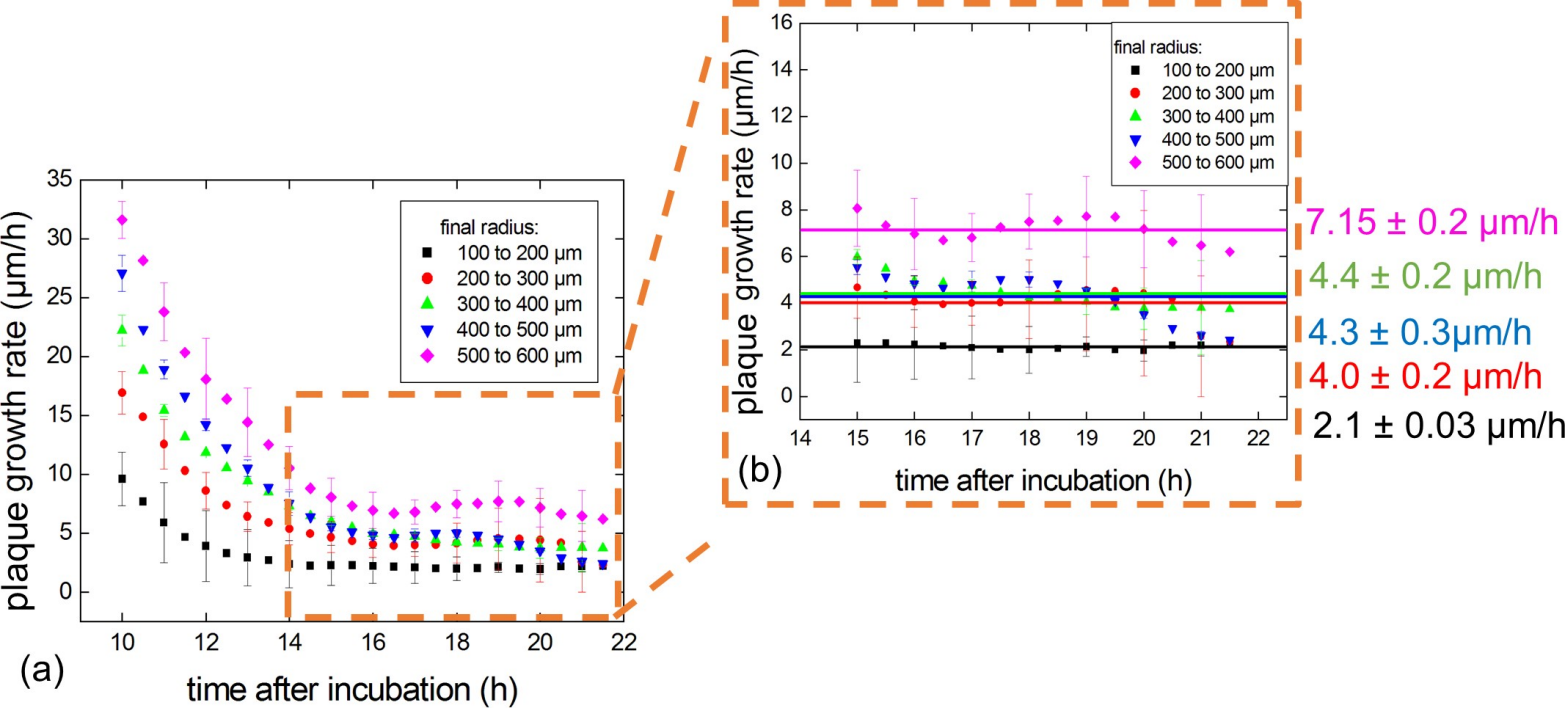
Study of plaque growth rates over time. Plaque growth rates vs time. (a) At 15 hr of incubation, the mean growth rate stabilized for all plaques independent of the size reached by the end of the experiment. (b) The mean growth rates during phase III were determined for each group.

During phase II, starting from 10 hr after the start of incubation, the plaque growth was no longer linear. Therefore, we analysed the growth rate of each plaque during phase II by computing the time derivative of the plaque radii (Fig 6A). For clarity, the plaques were classified into five different groups according to the size of their final radii. While plaque growth rate decreases for all groups, the plaque growth rate still correlates with the plaque size, being higher in larger plaques (Fig 7) as observed during phase I (see above). Therefore, we observed that despite a decrease in growth rate, larger plaques always grew faster than smaller plaques in both phase I and phase II.

In our experiments, the observed decreases in plaque growth rate during phase II could be correlated with the maturation of the bacterial lawn. Indeed, starting at 12 hr (i.e. at the beginning of phase II), the variation of the gray level of the bacterial lawn area was close to 0 hr^-1^ (Fig 5). This indicates that the amount of light transmitted through the bacterial lawn stopped decreasing as the bacteria ceased to divide. Therefore, our results fully support the previously reported observation that the plaque growth rate decreases as the host density increases [36].

In addition, hindered diffusion of phage particles in a dense bacterial lawn could contribute to the decrease in plaque growth rate during phase II. Indeed, the growth medium is not an homogeneous medium but rather a hydrogel (soft agar) which includes many bacterial microcolonies which may act as diffusional barriers [37].

Therefore, our results are in agreement with a general decrease in plaque growth rates in phase II resulting from impeded diffusion of the phages due to the high density of the bacterial lawn.

#### Phase III

Finally, after 15 hr of incubation (phase III), the plaque growth rates stabilized (Fig 6B). However–as in phase I and II–the plaque growth rates somehow correlated with the final plaque size as the largest size group (500 to 600 μm) had a mean growth rate of 7.15 ± 0.2 μm/hr while the smallest size group (100 to 200 μm) had a mean plaque growth rate of 2.1 ± 0.03 μm/hr. However, and interestingly, the three intermediary size group displayed similar plaque growth rates at around 4 μm/hr.

Therefore, our results suggest that the bacterial lawn reached full confluence at around 15 hr and that, despite consistently more rapid growth rates for larger plaques, the growth rates stabilized in such a homogeneous bacterial lawn.

### Imaging of phage-resistant microcolonies within plaques

**Fig 8:**
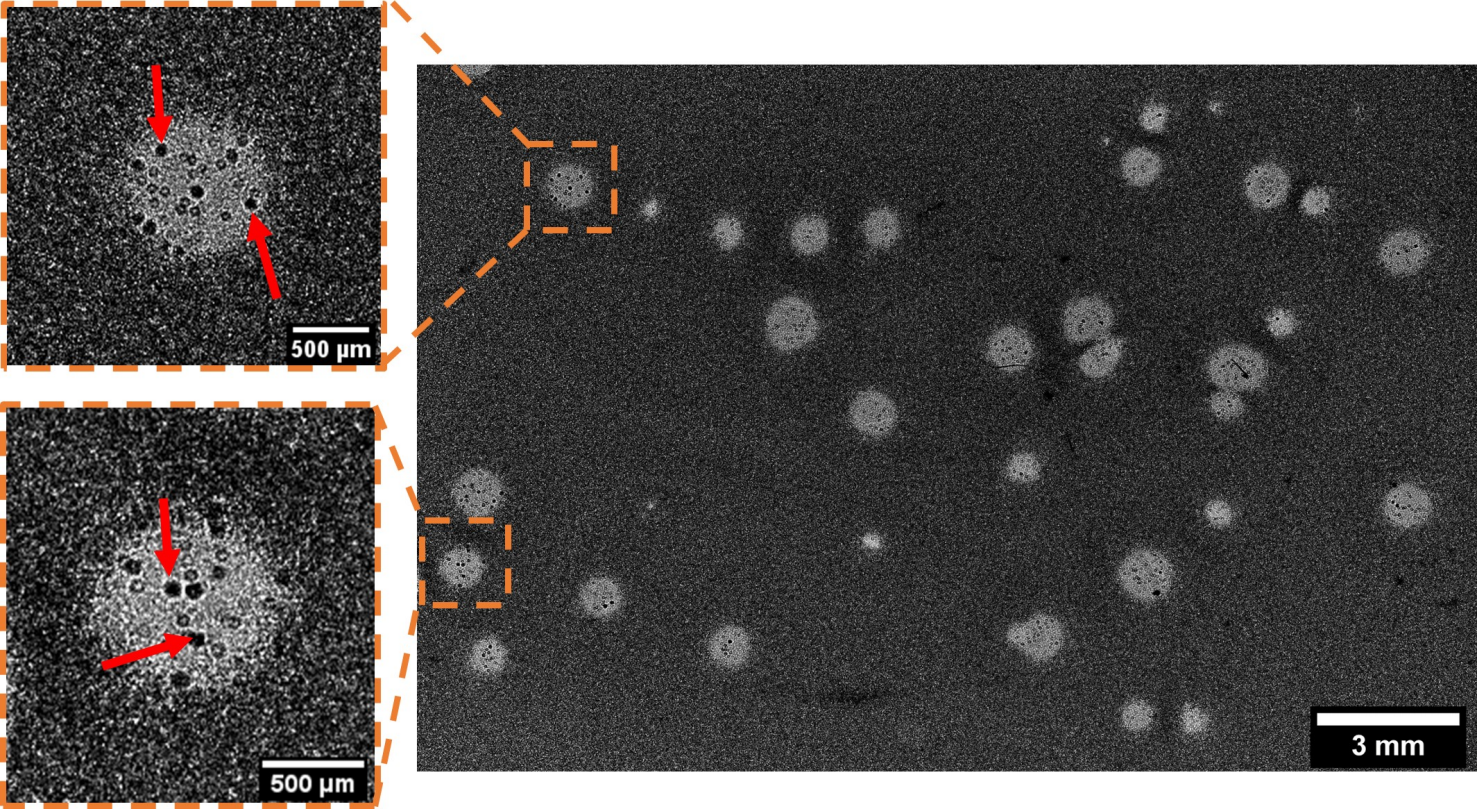
Lensless imaging of *K*. *pneumoniae* bacterial microcolonies developing within plaques after 23 hr of incubation. The red arrows indicate microcolonies within plaques.

Phage-resistant bacterial clones selected under phage pressure can develop as microcolonies within plaques [38, 39] and be imaged through lensless imaging (Fig 8). Indeed, the resolution of the sensor being limited to the pixel pitch (here 4.3 μm), we were able to image elements with sizes in the same order of magnitude. For instance, we imaged resistant microcolonies of *K*. *pneumoniae* with a diameter of 60 μm (Fig 8, red arrows). A movie of the acquisition is also available as a (S2 Movie).

These microcolonies probably represent *K*. *pneumoniae* bacteria that are resistant to phage vB_Kpn_5055, but this would have to be confirmed through isolation and reculturing of these colonies followed by a susceptibility test. However this result highlights that lensless imaging can be used as a preliminary tool to detect possible phage resistance to a bacterial strain. Indeed the wide 3.3 cm^2^ FoV coupled to the 4.3 μm pixel pitch enable the study of the evolution of millimeter-scale plaques while still permitting a resolution which is high enough to simultaneously visualize elements on the scale of around a few dozen micrometers.

## Conclusion

In this work, we report the use of a custom lensless device, over a 3.3 cm^2^ area sensor, for the continuous monitoring of phage lysis plaques. Leveraging wide-field lensless imaging, we performed a computer-assisted detection and counting of plaques that allowed detection of bacterial susceptibility to phages within 3 hr and accurate estimation of infectious titer in only 8 hr 20 min.

Moreover, by investigating the growth rates of 19 isolated plaques we confirmed the previously observed correlation between bacterial density and phage diffusion in the soft-agar layer. However, lensless imaging allowed us to identify three very distinct phases during the growth of a plaque independent of the size of the final plaque. Moreover, we demonstrated that plaques harboring the smaller sizes at the end of the experiment were always associated with a delayed onset of growth in phase I as well as lower growth rates during the three phases.

In addition, we showed that lensless imaging could be a powerful tool to screen the emergence of phage-resistant bacteria by imaging bacterial microcolonies within plaques. Additional experiments are needed to evaluate whether this technique could lead to an estimation of the phage-resistance frequency.

Future work will also focus on the development of an algorithm allowing morphological classification of plaques (and therefore phages) according to their plaque growth rates but also their morphotypes (though we did not discuss this aspect here). Moreover, we think that lensless imaging could reveal a very important approach to study phage-antibiotic synergy (PAS). Indeed, it has for instance been reported that the inclusion of an antibiotic in the agar layer leads to significantly larger plaques [6], making visible plaques that would otherwise be invisible to the naked eye [40]. Finally, a setup based on multiple image sensors working simultaneously is currently under development, which will increase the field of view and consequently the amount of data acquired at the same time.

## Supporting information

S1 Movie Lensless acquisition of single-layer soft agar assay of *S*. *aureus* strain Laus102 with lytic phage vB_SauM_2002. (AVI)

S2 Movie Lensless acquisition of single-layer soft agar assay of *K*. *pneumoniae* strain R405 TMP-8 and the lytic phage vB_Kpn_5055. (AVI)

S1 Dataset Phage plaque image stack from vB_SauM_2002 on *S*. *aureus* Laus102 acquisition. (ZIP)

S2 Dataset Measured phage plaque areas of phage vB_SauM_2002 on *S*. *aureus* Laus102 strain prior to processing. (CSV)

S1 File FIJI macro used to measure phage plaque kinetics. (ZIP)
