# Supplementary figures and images for: Phage susceptibility testing and infectious titer determination through wide-field lensless monitoring of phage plaque growth
Source: PLoS One. 2021 Mar 23;16(3):e0248917. doi: 10.1371/journal.pone.0248917 (PMC7987195; doi:10.1371/journal.pone.0248917)

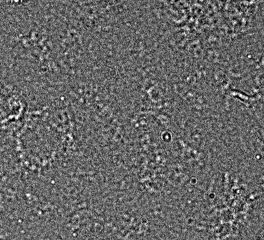

Supplement: S1 Dataset — (ZIP) [file pone.0248917.s003.zip › ref01_10min.tif]
